# The Pathology of Tumours

**Published:** 1917-07

**Authors:** 


**The Pathology of Tumours.** By E. H. Kettle, M.D., B.Sc. Pp. viii. 224. London: H. K. Lewis & Co. Ltd. 1916. Price 10s. 6d. net.

This is a very lucid and instructive book, and informs us as to the recent developments of the knowledge of the pathology of tumours. Throughout the book are very clear and beautiful drawings of the histology of tumours, and these constitute a marked feature of the work, and are very helpful. Some of the illustrations of tumours in museum jars are not clear, and might well have been omitted, but there are several pictures of the naked-eye appearance of tumours which are excellent.

Although the author says he has consulted standard text-books, special monographs, and articles in various scientific journals, he has purposely refrained from giving reference to the literature. This we regret, as it is always an advantage for the reader to be able to turn to writings of other authors on a subject in which he is interested. Perhaps the author of the book may add references to the literature in the next edition.

The chapter on the experimental study of cancer is particularly interesting and instructive, and gives very clearly the result of the transmission of cancer by inoculation in the mouse, and the lessons to be learnt from it.

In describing the characters of a tumour which may be regarded as indications of its malignancy, we think the author includes what we should regard as the essential characters of a malignant growth, such as a tendency to infiltrate, dissemination in glands, and other parts, and recurrence, when so far as the surgeon can judge by the naked eye he has completely removed it; but he also includes other characters, which though often present in malignant growths we should hardly regard as characteristic, such as ulceration, power of continuous growth, rapidity of growth, and tendency to central degeneration. These conditions might be also present in an innocent tumour. With regard to ulceration, it is admitted that it may be present in innocent tumours, and absent in malignant ones. Then why give it amongst the "accepted signs of malignancy"? And it certainly seems to us that it would not be wise to regard a tumour with central degeneration as necessarily malignant. As torapidity of growth being an accepted sign of malignancy, we must say we have seen some tumours, histologically quite innocent, grow rapidly, though of course rapidity of growth strongly suggests malignancy. Exactly what the author means by "power of continuous growth" is not evident, for obviously any innocent tumour has what we should usually understand by this. Possibly the author means power of continuous growth on transplantation.

The chapter on "Treatment" seems quite out of place in a book on the pathology of tumours, though it might be necessary to consider to some extent the pathology of tumours in a book on their treatment. But in this chapter there are some very interesting and instructive remarks by the author on the diagnosis of the nature of a tumour by immediate microscopic examination. The question as to what help the surgeon may really expect from the pathologist in this way is well considered, and the difficulty in making an immediate histological diagnosis, in some cases, is clearly set forth.

The very interesting and much discussed question of the transference of innocent into malignant growths is briefly considered from the pathological aspect, which is, we think, the only aspect which can afford conclusive evidence. The author considers that this transference is proved beyond all doubt from his own histological investigations. In connection with this subject, he makes the exceedingly interesting statement that he has found secondary malignant growths in an adenoma of the thyroid gland.

In the account of glioma we notice an unfortunate contradiction of the author's own information. He first says "gliomata are found only in the central nervous system and its out-growths, and the great majority occur in the brain." This seems quite definite, but on the next page we read that they occur outside the central nervous system, and "may grow in connection with the retina or the medulla of the suprarenal gland."

Amongst the innocent tumours of bone, we think cysts should find a place, as so much recent knowledge about them has now accumulated, and some information should be given as to their relation to so-called "osteitis fibrosis."

We feel obliged to point out some defects and omissions, but we can strongly recommend this book as a guide to the pathology of tumours.

